# Characterization of *Mycobacterium smegmatis* Glutaminase-Free Asparaginase (MSMEG_3173)

**DOI:** 10.1021/acsomega.4c06459

**Published:** 2024-09-12

**Authors:** Paloma Rezende Corrêa, Marcos Gustavo Araujo Schwarz, Deborah Antunes, Sindy Licette Piñero, Marlon Castro Silva, Mayra Mangabeira Crescêncio, Ana Carolina Ramos Guimarães, Wim Maurits Degrave, Leila Mendonça-Lima

**Affiliations:** Laboratório de Genômica Funcionale Bioinformática, Instituto Oswaldo Cruz, Fiocruz, Rio de Janeiro 21040-900, Brazil

## Abstract

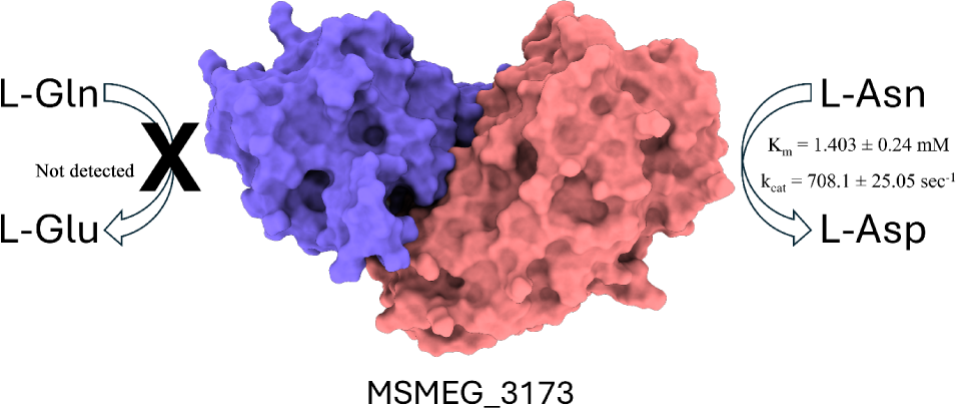

l-asparaginase is an enzyme catalyzing the hydrolysis of l-asparagine into l-aspartate and ammonia, which is of great therapeutic importance in tumor treatment. However, commercially available enzymes are associated with adverse effects, and searching for a new l-asparaginase with better pharmaceutical properties was the aim of this work. The coding sequence for *Mycobacterium smegmatis*l-asparaginase (MsA) was cloned and expressed. The recombinant protein showed high activity toward l-asparagine, whereas none was detected for l-glutamine. The enzymatic properties (*K*_m_ = 1.403 ± 0.24 mM and *k*_cat_ = 708.1 ± 25.05 s^–1^) indicate that the enzyme would be functional within the expected blood l-asparagine concentration, with good activity, as shown by *k*_cat_. The pH and temperature profiles suggest its use as a biopharmaceutical in humans. Molecular dynamics analysis of the MsA model reveals the formation of a hydrogen bond network involving catalytic residues with l-asparagine. However, the same is not observed with l-glutamine, mainly due to steric hindrance. Additionally, the structural feature of residue 119 being a serine rather than a proline has significant implications. These findings help explain the low glutaminase activity observed in MsA, like what is described for the *Wolinella succinogenes* enzyme. This establishes mycobacterial asparaginases as key scaffolds to develop biopharmaceuticals against acute lymphocytic leukemia.

## 1 Introduction

l-asparaginase (EC 3.5.1.1) is an enzyme catalyzing the deamidation of l-asparagine (l-Asn) into l-aspartate (l-Asp) and ammonia.^1^ It is normally also active toward l-glutamine (l-Gln) due to the structural similarity between the amino acids l-Asn and l-Gln, differing only in the presence of an additional methylene group on the side chain aliphatic portion of the latter.

The enzyme was first isolated in the beginning of the 20th century from different animal sources, and its biological role in the metabolism of l-Asn was elucidated.^2^ Its antitumor activity was discovered around 1950 when its impact on lymphoma growth regression and mice survival was evaluated.^3^ Since then, l-asparaginase has been used in different types of cancer treatment, including acute lymphocytic leukemia (ALL), T-cell lymphomas, ovarian cancer, and other solid tumors.^4^

l-Asn is an amino acid, essential for the growth of both tumors and healthy cells,^5^ but tumor cells usually have low or no expression of asparagine synthetase, hindering and/or inhibiting the *de novo* synthesis of l-Asn. Thus, ALL tumor cells need to obtain this amino acid from the extracellular environment to maintain their normal metabolism.^6^ The depletion of l-Asn in the bloodstream due to l-asparaginase activity causes nutritional restriction of certain tumor cell lines, inhibiting protein biosynthesis and triggering their apoptosis process. Notwithstanding, in healthy cells, asparagine synthase catalyzes the conversion of l-Asp and l-Gln into l-Asn and l-glutamate (l-Glu), respectively, through a transamination reaction, thereby maintaining the essential supply for cell metabolism.^5^

l-asparaginases from some microorganisms can hydrolyze just 5% of l-Gln when compared with the hydrolysis of l-Asn (100%), such as the enzyme from *Serratia marcescens*, *Escherichia coli*, and *Erwinia chrysanthemi*, while other l-asparaginases, such as those from *Pseudomonas* sp.^7,8^ and *Acinetobacter glutaminasificans*(9) show equivalent levels for both activities. The impact of glutaminase activity on antitumor treatment is still unclear, but there is evidence supporting that the main side effects observed during treatment with this biopharmaceutical are due to this “secondary” activity.^10^ Several studies focus on modifying native scaffold proteins to reduce l-glutaminase activity, maintaining l-asparaginase function. An interesting example is the enzyme from *Wolinella succinogenes* (WoA), in which mutation of a residue outside the dual catalytic triad, P121S, drastically reduces glutaminase activity without affecting asparaginase activity. Even outside the dual catalytic triad, it was shown that this residue, depending on the amino acid, aids in loop closure with l-Gln, therefore creating a proper catalytic site and increasing l-glutaminase activity.^11^

Additionally, the immune response of the patient to the biopharmaceutical can impact the treatment’s effectiveness. The dose and immunogenicity of the l-asparaginase administered can stimulate increasing antibody development against the enzyme through either intramuscular or intravenous administration. The main consequence of this reaction is the rapid decline in l-asparaginase concentrations after its administration.^12,13^

Although some l-asparaginases are naturally available, they are generally not recognized as suitable for therapeutic purposes due to the high cost of production, adverse effects, and the risk of patient hypersensitivity.^14^ The search for new sources of l-asparaginase is essential to provide more and better options on the market, whether as such or as a scaffold for genetic improvement, focusing on better biochemical features and fewer side effects. l-asparaginase activity was described for several species of the genus *Mycobacterium*, including *M. tuberculosis* (M. tb), *M. bovis*, *M. lacticola,* and *M. smegmatis*.^15^ Inhibition of mice sarcoma growth was achieved using a lower concentration of the M. tb enzyme when compared to the required concentration of *E. coli*l-asparaginase.^16^ Furthermore, it was observed that M. tb and *M. phlei*l-asparaginases have high asparaginase activity, but undetectable glutaminase activity.^17^ In the present study, the gene encoding the l-asparaginase from *M. smegmatis*(MsA=Uniprot A0QX50) was cloned and expressed in *E. coli* BL21 (DE3). The recombinant enzyme was purified and the resulting product was biochemically analyzed. Comparative structural modeling and molecular dynamics analyses were used to elucidate its mechanisms, aiding to explain the low glutaminase activity and also enabling its future optimization through the identification of possible T- and B-cell epitopes more accessible to the solvent, that can be further mutated to decrease immunogenicity of this potential biopharmaceutical.

## 2 Materials and Methods

### 2.1 Cloning, Expression, and Purification of Recombinant MsA

For recombinant MsA production, the sequence corresponding to *MSMEG_3173* (position 3248 594.3249553 on *M. smegmatis* mc^2^ 155 genome) was PCR amplified and cloned in pET28a(+), adding an N-terminal 6-His tag to the final recombinant protein. *M. smegmatis* mc^2^ 155 genomic DNA was used as a template to amplify the fragment containing a NdeI restriction site 5′ overhang and a *Hin*dIII restriction site 3′ overhang, using Platinum SuperFi DNA polymerase (Invitrogen) and the following pair of primers: AGCCATATGAACCCGTCCGCACAAGTC (forward) and ACTAAGCTTTCAACCCCATGAGGAGAAAAC (reverse).

After selection of the desired DNA construct in *E. coli* TOP10 and sequence confirmation, plasmid DNA was transformed into *E. coli* BL21(DE3) for recombinant protein expression. Cells were harvested after induction with 1 mM isopropyl-β-D-thiogalactopyranoside (IPTG) for 3 h at 37 °C, in Luria–Bertani (LB) medium containing 25 μg/mL kanamycin and 1% (w/v) glucose. A bacterial pellet was resuspended in lysis buffer (20 mM Tris-HCl pH 7.5; 300 mM NaCl; 0.5% [v/v] Triton X-100) and disrupted by mechanical lysis on a BeadBeater equipment using glass beads and 3 × 1 min cycles on ice.

His-tagged protein was purified by Immobilized Metal Affinity Chromatography (IMAC). The resulting supernatant after lysis was clarified by centrifugation and 0.22 μm filtered before being loaded on a HisTrap HP 1 mL column (GE Healthcare), charged with Ni^2+^. Loading buffer (buffer A) was 100 mM Tris-HCl pH 7.5, 300 mM NaCl and 5 mM imidazole. Elution was performed by 5 column volumes (CV) step-gradient (10, 20, 30, 40, and 100%) with elution buffer (same as loading, but with 500 mM imidazole). Fractions were analyzed by 12% SDS-PAGE, gels were stained with Coomassie Brilliant Blue *R*-250 (CBB-R250) and those fractions containing purified protein were pooled. Purified protein was concentrated, and buffer was exchanged (200 mM Tris-HCl pH 7.5; 20 mM NaCl) by centrifugation in 30 kDa centrifugal filter devices (Amicon Ultra, Millipore). Throughout all analyses, protein concentration was assessed using a Nanodrop equipment by 280 nm absorbance measurements.

### 2.2 Enzyme Activity Assays

Enzyme activity was measured as previously described.^18^ In standard assays, enzyme solution was added to a 1.05 mL reaction mixture containing 9 mM substrate in 50 mM Tris-HCl, pH 8.6 buffer. After 30 min incubation at 37 °C, reaction was stopped by addition of trichloroacetic acid (final concentration ∼0.07 M). Then, 100 μL of the reaction mixture was diluted with 2.15 mL of Milli-Q water, followed by the addition of 250 μL of Nessler’s reagent, and the solution was incubated for at least 1 min. The amount of NH_3_ released and enzyme activity was calculated by measuring absorbance at 436 nm. A standard curve was prepared with ammonium sulfate. One international unit (U) is defined as the amount of enzyme producing 1 μmol of NH_3_ in 1 min at 37 °C. Specific activity is the amount of enzyme units per protein mass in mg.

To analyze the enzyme substrate specificity, standard assays with l-Asn or l-Gln were performed. In this experiment, Leuginase,^19^ a commercial *E. coli*l-asparaginase 2 preparation, was used for comparison.

To assess the effect of pH and temperature on enzyme activity, assays were performed in different buffers (200 mM sodium acetate [pH 3.5, 4.0, 4.5, 5.0 and 5.5], sodium citrate [pH 5.5 and 6.0], sodium phosphate [pH 6.0, 6.5, 7.0, 7.5 and 8.0], or Tris-HCl [pH 8.0, 8.5 and 9.0], at constant 37 °C) or temperatures (25, 30, 37, 40, 45, 50, 55, 60, or 65 °C, at constant pH8.6).

To calculate enzyme kinetic parameters (*K*_m_ and *k*_cat_), initial velocity (mM/s) was measured at different l-Asn final concentrations (0.05, 0.2, 0.4, 0.8, 1.0, 2.0, and 4.0 mM). Data were fitted by nonlinear regression analysis of steady-state rate using GraphPad Prism 5 software. All results are given as the mean ± standard deviation of a set of three independent experiments.

### 2.3 Prediction of B and CD4+ T Cells Epitopes in MsA

The MsA protein primary sequence was used to assess possible B and CD4^+^T cell epitopes, as well as the immunogenicity score calculated by online tools, such as the Immune Epitope Database by the CD4 T cell immunogenicity prediction tool (http://tools.iedb.org/CD4episcore/.) and the Artificial neural network based B-cell epitope prediction server (ABCpred, https://webs.iiitd.edu.in/raghava/abcpred/index.html).

### 2.4 Comparative Modeling

The homotetrameric structure of MsA (MSMEG_3173) was modeled using the Robetta^20^ comparative modeling method with *Wolinella succinogenes*l-asparaginase (WoA) as a structure template (PDB ID: 5K3O.^11^ The best constructed model from the output of the Robetta server was selected for additional evaluation and model validation.

The resulting structure was minimized using the UCSF Chimera.^21^ Protein interactions were represented using amber ff14SB force-field.^22^ A short minimization procedure of 110 steps (100 steps of steepest descent plus 10 steps of conjugate gradient) was performed. Initial and optimized models were evaluated by the structure assessment tool of the Swiss-Model server.^23^

### 2.5 Molecular Docking

Molecular docking assays were performed between the ligand l-Asp and the MsA model. Calculations were carried out using the DockThor^24^ web service with default parameters: number of evaluations “1,000,000″, population size “750″, initial seed “–1985” and a number of runs “24”. The search space coordinates were center *X* = 24.917, *Y* = 2.274, *Z* = 64.757, and its dimensions *X* = 15000, *Y* = 15000, *Z* = 15000, with a discretization of 0.25. Before simulations were carried out using the modeled MsA, a redocking protocol was established using the WoA complex with l-Asp (PDB ID: 5K3O) to validate Dockthor performance. The 2D-interaction maps were generated using Discovery Studio Visualizer 2021^25^ and a three-dimensional structure figure with UCSF Chimera.^21^

### 2.6 Molecular Dynamics

MD simulations were carried out using AMBER 20,^26,27^ and protein interactions were represented using amber ff14SB force-field.^22^ Bonded, electrostatic, and Lennard–Jones parameters for the l-Asp were obtained using the generalized amber force field (GAFF)^28^ and AM1-BCC^29^ tools. The atomic partial charges were added using ANTECHAMBER.^30^ Electrostatic interactions were treated using the particle-mesh Ewald (PME) algorithm with a cutoff of 12 Å. Protonation states of titratable residues were assigned using PDB 2PQR server^31^ considering the pH of 7.0. Each system was simulated under periodic boundary conditions in a triclinic box, whose dimensions were automatically defined, considering 10 Å from the outermost protein atoms in all Cartesian directions. The simulation box was filled with TIP3P water molecules.^32^

Subsequently, a two-step energy minimization procedure was performed: (i) 2000 steps (1000 steepest descent + 1000 conjugate-gradient) with all heavy atoms harmonically restrained with a force constant of 5 kcal mol^–1^ Å^–2^; (ii) 5000 steps (2500 steepest descent + 2500 conjugate-gradient) without position restraints. Next, initial atomic velocities were assigned using the Maxwell–Boltzmann distribution corresponding to a temperature of 20 K. The systems were gradually heated to 300 K over 1 ns using the Langevin thermostat. All heavy atoms were harmonically restrained during this stage with a force constant of 10 kcal mol^–1^ Å^–2^. All systems were subsequently equilibrated during nine successive 500 ps equilibration simulations, where position restraints approached zero progressively. After this period, all the systems were simulated with no restraints at 300 K in the Gibbs ensemble with a 1 atm pressure using isotropic coupling. All chemical bonds containing hydrogen atoms were restricted using the SHAKE algorithm^33^ and the time step was set to 2 fs. We simulated three independent MD runs of 200 ns for MsA and WoA complexes using different initial velocities.

### 2.7 Trajectory Analysis

Simulation trajectories were analyzed with GROMACS package tools.^34^ Root-mean-square deviation (RMSD) values and solvent-accessible surface areas (SASA) were calculated separately for each system, fitting their backbone atoms, taking the initial structure of the production dynamics as a reference. Conformational clusterization for protein was performed using the GROMOS method with a cutoff of 2.0 Å, considering all atoms from the last 50 ns trajectory of each MD simulation. The central structure of the largest cluster was taken for electrostatic potential analysis. The Amber force field charge and radii parameters were assigned using the PDB 2PQR server,^31^ considering the pH values of 5, 7, and 9.

Hydrogen bonds (H-bond) were calculated for each system, concatenating three independent MD simulations’ last 50 ns trajectory. H-bond formation was defined using a geometric criterion with CPPTRAJ^35^ in Amber. We considered a hit when the distance between two polar heavy atoms, with at least one hydrogen atom attached, was less than 3.5 Å, and using an H-donor angle higher than 120°.

Principal component analysis (PCA) was carried out for all systems using Bio3D^36^ library as implemented in R.^37^ Rotational and translational motions were removed before calculation of the covariance matrix by least-squares superposition to the corresponding average structures. The 3N × 3N covariance matrices of *x*-carbon atomic positions (Cartesian coordinates) were then calculated for each system. The conformations explored during the MD simulations were applied using hierarchical clustering in R (hclust) with the complete linkage method based on the PC1–PC2 subspace, where PC1 and PC2 denote the projections onto the two first eigenvectors.

We analyzed the protein energy network (PEN) between pairs of residues to investigate how l-Asp binding affects the energy couplings in MsA and WoA. The PEN was performed with gRINN tool,^38^ by taking each residue in the structure as a node and determining edges and edge weights/distances using average interaction energies (van der Waals and electrostatic). A PEN was constructed for MsA and WoA systems by extracting 1500 snapshots from the last 50 ns trajectory of each MD simulation, excluding covalent bonds as edges and adding edges between any two residues whose absolute average interaction energy were equal to or above 1 kcal/mol.

## 3 Results

### 3.1 Recombinant MsA Purification

**Figure 1:**
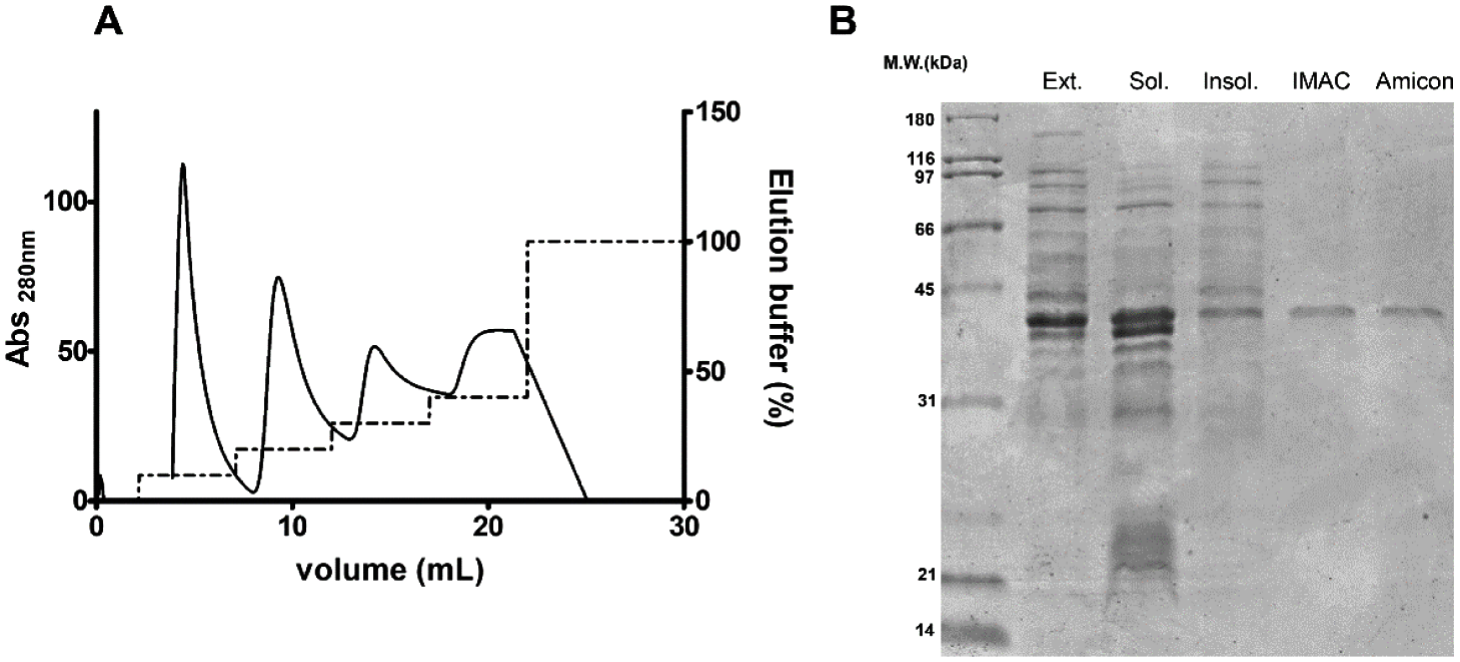
Recombinant MsA purification steps. After inducing the proper clone, cells were mechanically disrupted, and total extract (Ext) was clarified by centrifugation, leading to the soluble (Sol) and insoluble (Insol) fractions. The former was used in a Ni^2+^-IMAC, leading to a purified MsA sample in the 20% elution buffer step (A). The latter was further concentrated and buffer exchanged (Amicon). The overall purification was also assessed by analyzing protein complexity in all samples by 12% SDS-PAGE, stained with CBB-R250 (B).

We successfully cloned the PCR amplified 963 bp gene fragment (*MSMEG_3173*) in the pET28a(+) NdeI/*Hin*dIII cloning sites. The resulting recombinant protein has a N-terminal 6-His tag and an estimated monomer molecular weight and pI of 34.5 kDa and 5.98, respectively. The enzyme activity and total protein content was assessed throughout the purification process to determine purification fold and yield, shown in Table 1. Under the assay conditions, this polypeptide was largely produced in a soluble form and purified by Ni^2+^-IMAC (with a purification fold of 100.5), mostly eluted in the 20% elution buffer step. As shown in Figure 1, after IMAC the protein sample appeared as a single band with the expected molecular weight on SDS-PAGE analysis. This sample was further concentrated, leading to the working protein solution, with an overall purification fold of 186.8 compared to the total extract, with a 15.3% yield. Albeit the major purification step, IMAC also showed higher activity loss, as shown by the yield decrease (Table 1).

**Table 1:** Purification Table^a^

			Purification (fold)		
	activity (U/mL)	specific activity (U/mg)	step	global	yield (%)
Extract	4.95	0.25	--	1	100
Soluble fraction	3.64	0.20	0.8	0.8	82.3
Ni^2+^-IMAC	3.86	20.05	100.5	81.7	23.8
Amicon	12.62	45.85	2.3	186.8	15.3

a Throughout the overall process, extract, soluble fraction, Ni^2+^-IMAC purified and Amicon samples were analyzed to assess asparaginase activity and total protein concentration, allowing for the calculation of the specific activity, purification fold, and yield of each step.

### 3.2 Substrate Specificity and Enzyme Kinetics

**Figure 2:**
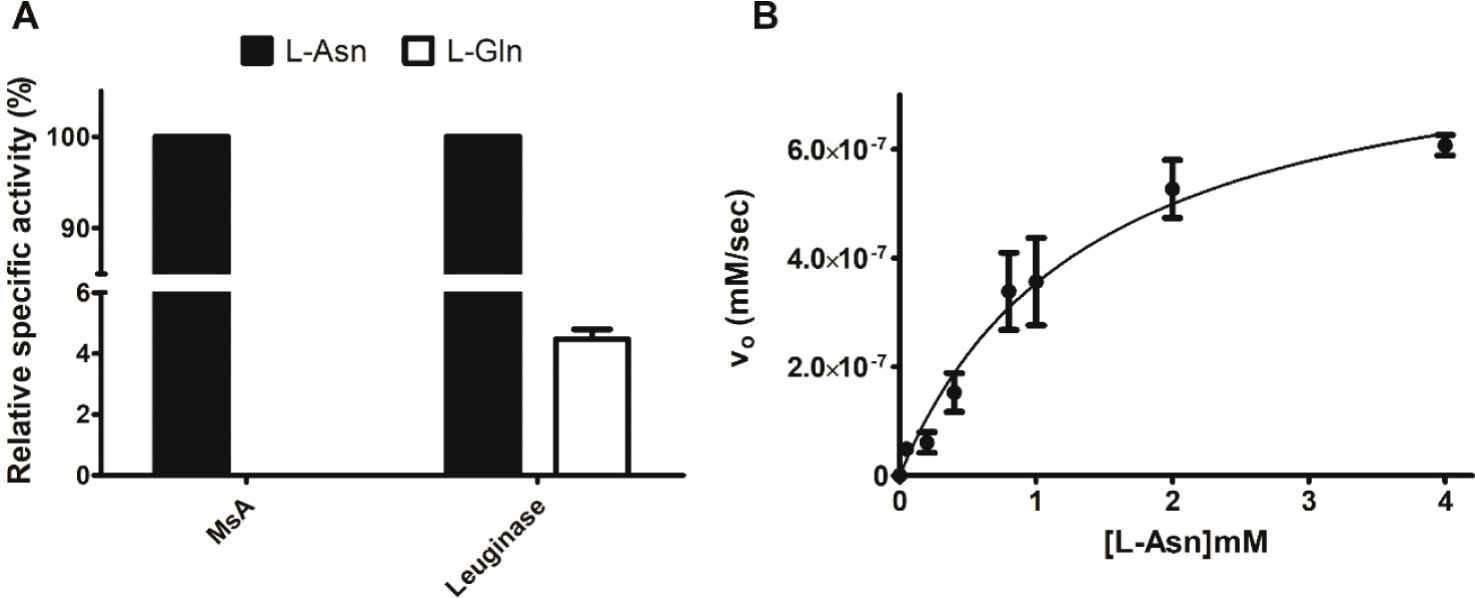
MsA substrate specificity and enzyme kinetics toward l-Asn. (A) Specific activity (U/mg) for MsA and Leuginase (a commercial *E. coli*l-asparaginase 2 preparation) was measured toward l-Asn and l-Gln. Activity using l-Asn as substrate was considered as 100%. (B) Initial velocity (mM/s) using l-Asn as substrate was measured in a range of concentrations (0.05 up to 4 mM). Data were fit in a nonlinear regression, and *K*_m_ and *k*_cat_ values were calculated by applying the created model. Results are represented as the mean ± the SD of a set of three independent experiments.

To assess MsA substrate specificity, we measured enzyme activity toward two substrates, l-Asn and l-Gln. For comparison, we used a commercial *E. coli*l-asparaginase 2 (EcA2) preparation (Leuginase). EcA2 possesses about 2% of glutaminase activity compared to asparaginase.^39^ In order to detect the percentage of glutaminase toward the overall asparaginase activity, we considered each specific activity toward l-Asn as 100% and glutaminase proportional to it. As can be observed in Figure 2A, Leuginase, under the assay conditions, showed about 4 ± 0.3% glutaminase activity. In contrast, it was not possible to detect any activity toward l-Gln in MsA, corroborating that this enzyme can be considered a glutaminase-free asparaginase.

Analysis of the MsA kinetic parameters toward l-Asn (Figure 2B) indicates a lower *K*_m_ value when comparing to other reported mycobacterial asparaginases, like the ones from *M. tuberculosis* (MtA) and *M. gordonae*. However, *k*_cat_ values were similar when comparing MsA and MtA (Table 2).

**Table 2:** MsA Kinetic Parameters Toward l-Asn, Compared to Published Values for other Mycobacterial and Commercial Bacterial Asparaginases^a^

	Asparaginase		Glutaminase		
	*K*_m_ (mM)	*k*_cat_ (sec^–1^)	*K*_m_ (mM)	*k*_cat_ (sec^–1^)	REF
*M. smegmatis* (MsA)	1.4 ± 0.2	708.1 ± 25.05	ND		this study
*M. tuberculosis* (MtA)	8.3 ± 0.5	869.4 ± 30.24	ND		(42)
*M. gordonae* (GmASNase)	6.025	197.7	ND		(43)
*E. coli* type 2 (EcA2)	0.02	440	1.67	65	(48)
*E. chrysanthemi* (ErA)	0.05	207	0.36	26.84	(47)

a ND: not detected under the assay conditions.

### 3.3 Characterization of MsA Structure and Its Binding Mode with Substrate

**Figure 3:**
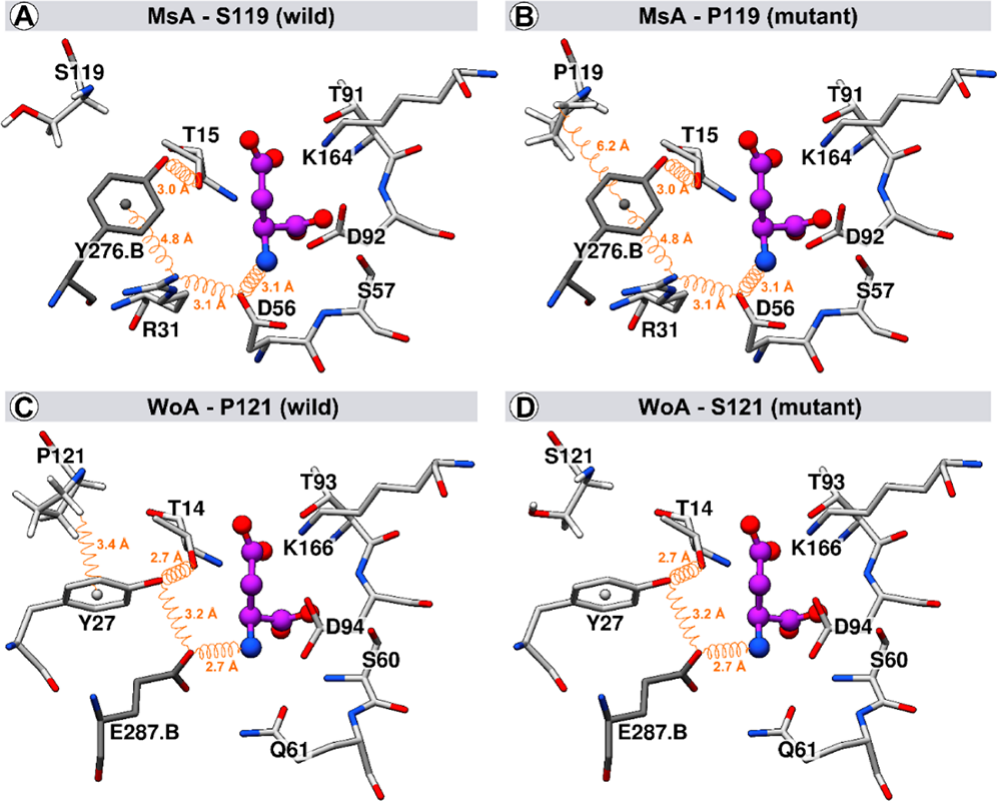
Three-dimensional structure of the catalytic sites of MsA and WoA. Pose of l-Asp (represented by the red [oxygen], purple [carbon], and blue [nitrogen] dots) in the MsA site derived from docking assays (A) and the WoA was taken of PDB ID: 5K3O (C). Also represented are mutations S119P in MsA (B) and P121S in WoA (D).

To assess the structural properties and binding mode of l-Asp with MsA, comparative modeling was employed. The crystal structure of *Wolinella succinogens*l-asparaginase (WoA), sharing 31% sequence identity and 97% sequence coverage with MsA (Figure S1), served as the template for constructing the model. Prior to optimization, the model’s structural quality was evaluated (Table S1), and subsequent optimization improved its quality, rendering it compatible with the template evaluation and suitable for molecular docking assays. Molecular docking was performed to predict the binding mode of l-Asp in the MsA model. To validate the docking protocol, cocrystallized l-Asp in WoA (PDB ID: 5K3O) was used as a reference. The docking experiments successfully reproduced the crystallographic position of l-Asp from 5K3O, achieving an RMSD of 0.208 Å (Figure S2). Building upon this success, the binding mode of l-Asp in the MsA model was explored by evaluating the three highest-scoring docking poses. The optimal result displayed an RMSD of 0.768 Å compared with the crystallographic position of l-Asp (Figure 3A,C).

The modeling elucidated the structural properties and l-Asp binding mode of MsA, unveiling the composition of catalytic triads and the presence of native serine at position 119 (MsA-S119). Triad II in MsA (T91, D92, and K164) closely resembles WoA, while in triad I, MsA-T15 is conserved and residue substitutions occur for WoA-Y27 (replaced by MsA-Y276.B) and WoA-E287.B (replaced by MsA-D56) (Figure 3). As described in WoA, l-Asn binding at the active site induces a hydrogen bond network, facilitating closure of the N-terminal loop containing residues E287.B, Y27, and T14. In MsA, the network involves l-Asp–D56–R31–Y276.B and T15 interactions, with R31 playing a crucial role (Figures 3A,B and S3). The proline at position 121 in WoA contributes to loop closure through a CH/π interaction between hydrogen δ 1 in P121 and the aromatic ring of Y27. However, this interaction is absent when serine is present at position 119 (Figures 3C,D and S4). Molecular dynamics simulations confirmed the stability and implications of MsA-S119P (equivalent to WoA-P121S) and MsA-R31.

**Figure 4:**
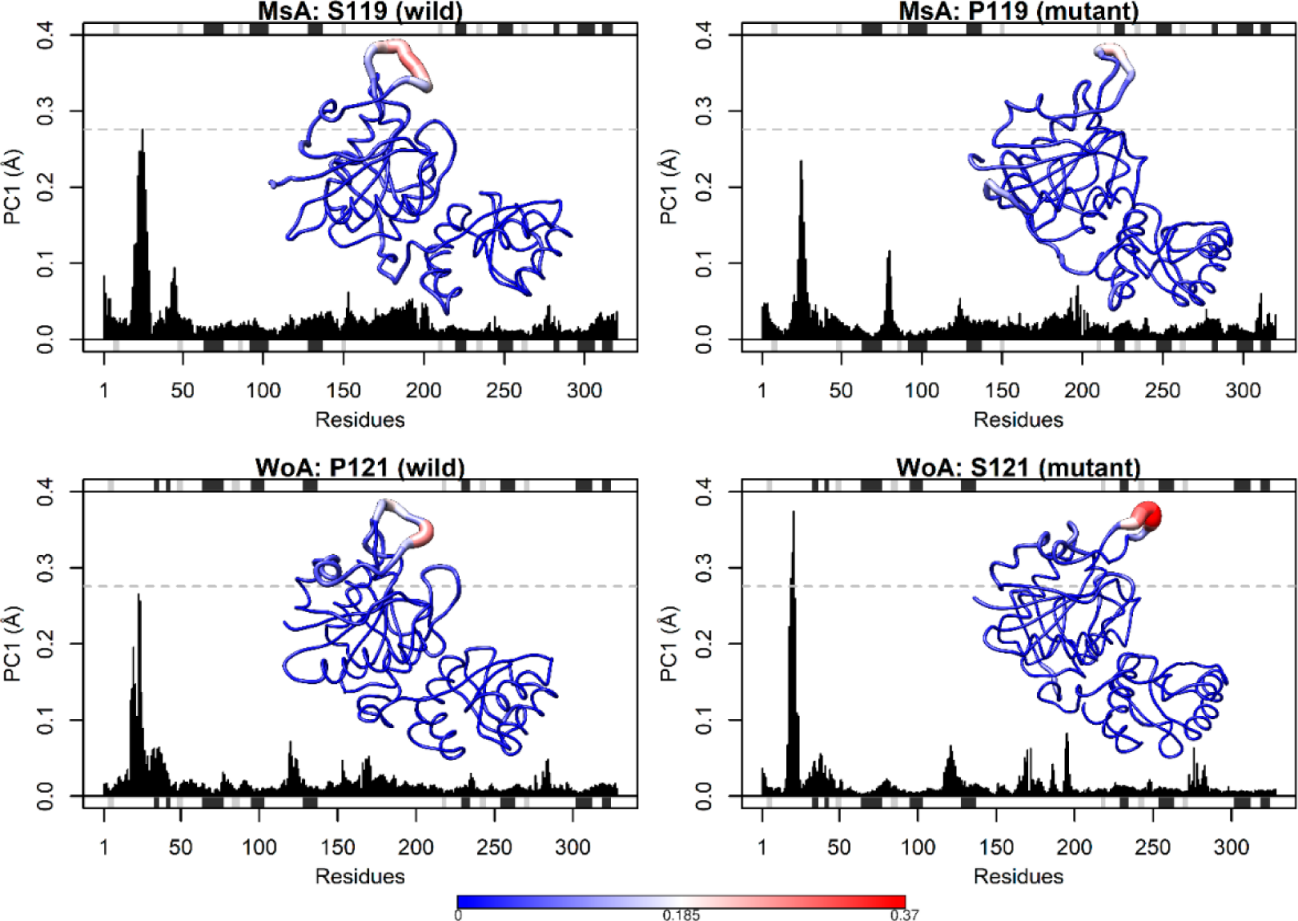
Root-mean-square fluctuation (RMSF) contributed by the first principal component (PC1). Structural representation obtained by displacements along with PC1 is displayed within each plot. Blue shows overlapping regions with little or no motion. Red areas represent mobile regions.

PCA revealed increased flexibility in the N-terminal loop of MsA-S119/WoA-S121 systems compared to their proline counterparts, indicating that serine reduces the loop stability (Figure 4). Interaction energy calculations highlighted the significance of MsA-T15/WoA-T14, with strong interactions below −1.4 kcal/mol. The interaction between MsA-Y276.B/WoA-Y27 and other residues also demonstrated the importance of R31 in MsA (Figure S5).

Furthermore, we investigated the binding mode of l-Asn and the potential steric hindrance of l-Gln within the catalytic site of MsA, building upon previous findings in WoA. Notably, the interaction network of l-Asn was found to be similar between WoA with P121 and wild-type MsA with S119 (Figure S6). Moreover, steric hindrance of l-Gln was observed within the catalytic triads of both WoA and MsA, suggesting comparable mechanisms of action for these systems (Figure S7). Thus, the elucidation of molecular events accounting for WoA’s glutaminase-free feature can be extended to wild-type MsA.

### 3.4 Effect of pH and Temperature on Asparaginase Activity

**Figure 5:**
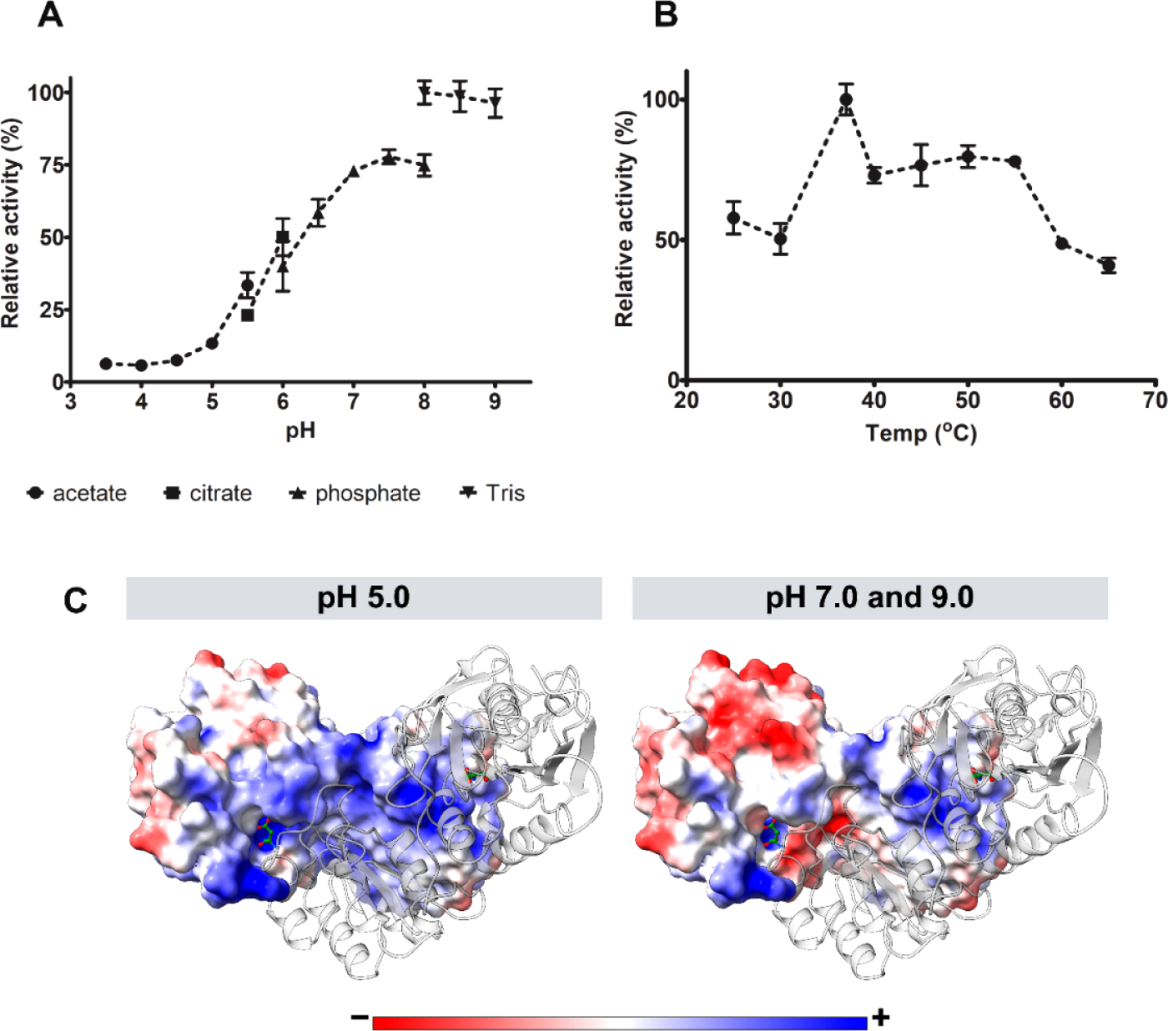
Effect of reaction pH and temperature on MsA asparaginase activity. (A) Asparaginase activity was measured at different pH values. To assess this, several buffers were used (when the buffer salt was exchanged, the same pH value was tested for each composition). (B) Activity was also assessed at different reaction temperatures, at pH 8.6. Values are relative to the higher activity obtained for each assay. Results are represented as the mean ± SD of a set of three independent experiments. (C) Surface electrostatic potential distribution on MsA at different pH values (5.0, 7.0, and 9.0). The surface is color-coded based on the electrostatic potential, with red, white, and blue representing acidic, neutral, and basic potentials, respectively.

To further biochemically characterize MsA, asparaginase activity was measured under different pH. To minimize buffer salt interference in observed values, when exchanging buffer, the same pH was tested for both. As shown in Figure 5A, MsA displays higher asparaginase activity toward basic pH, peaking around 7–8. The activity was also measured at different temperatures, reaching a maximum at 37 °C (Figure 5B).

To examine the effect of pH on the structure of MsA, the central structure from the predominant cluster of molecular dynamic simulations was analyzed. Protein charge was found to be pH-dependent, with a charge of 9.0 at pH 5 and −12.0 at pH 9 (Figure 5C). Furthermore, the distribution of the electrostatic potential on the surface of MsA was investigated. Notably, the active site of MsA exhibited a region of negative electrostatic potential at pH 7 and 9. Conversely, at pH 5, protonation of aspartic acid and glutamic acid residues (neutral) led to the formation of an extensive region of positive electrostatic potential.

### 3.5 Antigenic Epitope Mapping

To investigate the possible MsA immunogenicity in humans, a search of B and CD4+ T cell epitope databanks was performed. As shown in Table 3, B-cell epitopes are distributed around the N-terminal and the middle of the 320 amino acid length protein, whereas CD4+ T-cell epitopes are concentrated in the C-terminal region. It is also worth noting that almost all epitopes identified *in silico* are from B-cells (∼66%).

**Table 3:** B and CD4 T-cell Epitopes Identified in MsA^a^

Sequence		
B-cell epitopes	Position	Immunogenicity score
DGVVITHGTDSLEETALWLE	83..102	0.89
ALWLELTHTGDTPVVITGAQ	98..117	0.87
ASPDARGLGVLVCFAGTVRA	139..158	0.87
SDWDAIREAVAKATDGPGGP	63..82	0.81
GTISTSTDNAGVRRPTRRGA	14..33	0.8
NLADAIAVAASPDARGLGVL	130..149	0.8
CD4+ T-cell epitopes		
LGVLVCFAGTVRAPL	146..160	94.5268
RAGVEIVVSTRVPGG	256..270	91.9076
PQARVLLMAALAAQR	296..310	95.8981

a Individual epitopes are shown as linear peptide sequences along with their position in the MsA primary sequence and immunogenicity score (according to each prediction software).

**Figure 6:**
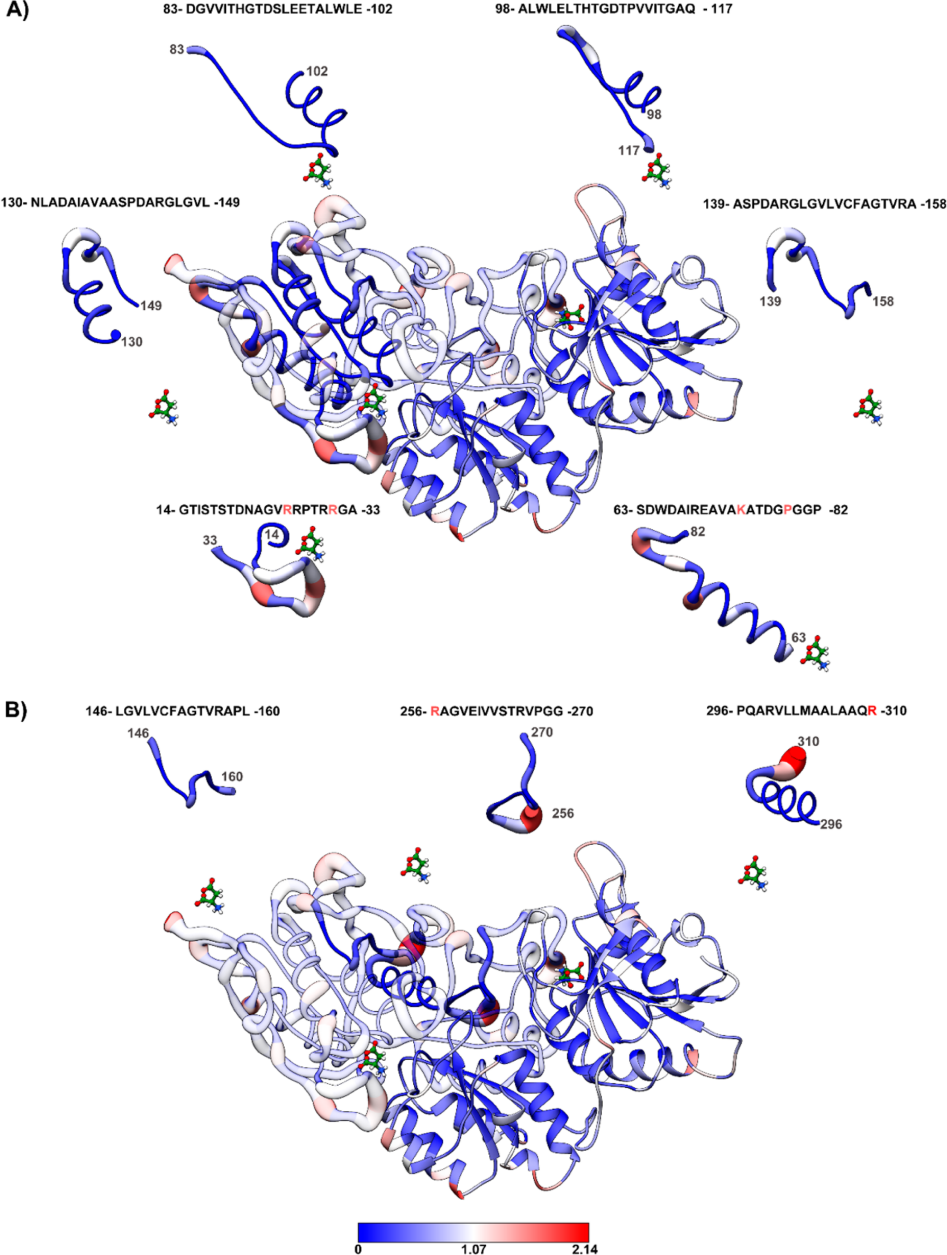
Three-dimensional representation of the residue solvent accessibility for each residue of MsA systems. Around the central structure, the regions containing the predicted epitopes of the B cell (A) and CD4+T cell (B) are highlighted. Each residue was colored according to the figure caption.

The residue solvent accessibility of MsA was analyzed by using molecular dynamics trajectories to identify predicted epitope locations (Figure 6). One notable epitope of interest encompassed residues 14 to 33, including T15 of triad II and R31 (Figure 6A). R31 exhibited a higher solvent-accessible surface area (SASA) value of 0.92 nm/S2/N, and R26 had a SASA of 1.58 nm/S2/N. Another solvent-exposed epitope within the region from residue 63 to 82 was identified, with K74 and P79 highlighted due to their SASA values of 1.59 and 1.61 nm/S2/N, respectively. In addition, the two CD4+ T-cell epitopes, characterized by R256 and R310, displayed the highest SASA values of 1.89 and 2.14 nm/S2/N, respectively (Figure 6B).

## 4 Discussion

Asparaginases have been the subject of mycobacterial research since the early 1950s. The main question at that time was how these bacteria employed l-Asn as a nitrogen source in a defined medium used for laboratory cultivation, such as the Sauton medium.^40^ Since then, the discovery of asparaginase activities throughout mycobacteria, including *M. bovis* BCG (Bacillus Calmette–Guérin), *M. phlei*, *M. butyricum*, virulent *M. bovis*, *M. lacticola,* and *M. smegmatis*, showed that this enzyme provides a widespread strategy to utilize nitrogen, also supplying α-keto-acid to different cellular metabolisms, such as the Krebs cycle anaplerotic reactions.^15^ The discovery of asparaginase activity in M. tb showed that the avirulent H37Ra strain possesses two different activities with distinct biochemical profiles, whereas the virulent H37Rv showed a single one.^41^ Interestingly, one of the M. tb H37Ra asparaginase inhibits the growth of Yoshida ascite sarcoma in rats at significantly lower concentrations than *E. coli* type 2 asparaginase, the major source of this enzyme for clinical trials at the time.^16,40^ Besides that, all tested mycobacterial asparaginases showed no detectable glutaminase activity.^15^

However, after this initial interest in mycobacterial asparaginase, little progress was made in the characterization of these enzymes, particularly focusing on their use as biopharmaceuticals in ALL treatment. In the field of tuberculosis therapeutics, secreted MtA appears to be an interesting drug target, as it promotes long-term bacilli survival through neutralization of the phagolysosome environment by ammonia liberation after l-Asn hydrolysis. It also induces stress to primary immune cells, compromising the host immune response, since MtA harbors several B and CD4+ T cell epitopes in its primary sequence.^17,42^ Mycobacterial asparaginases are also used in the food industry, where the *M. gordonae* enzyme is being tested to minimize acrylamide formation on the food frying process.^43^ Despite the use of these enzymes, all reports on biochemical characterization of mycobacterial l-asparaginases corroborate their natural glutaminase-free feature and measure enzyme kinetic parameters toward l-Asn, facilitating comparisons. Here, we report the biochemical characterization of MsA, and through *in silico* analysis offer an explanation for the absence of glutaminase activity.

Recombinant MsA was purified using IMAC yielding a global purification fold of 186.8, a higher value when compared to that described for the *M. gordonae* recombinant asparaginase (31.29). However, the yield was around 3 times lower (15.3% to 42.71%), which can be due to the smaller number of purification steps used in their work.^43^ Another possible explanation is the incomplete adsorption of the enzyme onto the resin under the tested purification conditions or partial denaturation, leading to a loss of enzymatic activity. These chromatographic steps will be adjusted in future studies to achieve higher yield levels. Additionally, optimization of *MSMEG_3173* codon usage for *E. coli* expression can increase protein production during the IPTG-induction step, since this mycobacterial gene has a high GC% content of 73.8%, higher than the usual 50% of the *E. coli* genome. Codon usage optimization minimizes rare codons, theoretically accelerating and stabilizing the translation process and optimizing recombinant protein expression, often favoring solubility.

Developing novel l-asparaginases into new and promising therapeutic products requires focus on critical enzyme features that make them competitive products in the already existing asparaginase market. Among all the desirable characteristics, three have been the focus of this work: (i) **enzyme kinetic parameters** adequate to physiological l-Asn (and l-Gln) serum concentration, as well as a good activity to achieve the desired l-Asn blood depletion; (ii) **low immunogenicity**, leading mainly to an increased half-life and sparser drug application intervals; and (iii) **low or zero glutaminase activity**. Here, we investigated these three parameters for recombinant MsA.^44^

First, as a biopharmaceutical working in the bloodstream, higher asparaginase activity should be achieved in the blood physicochemical environment. We demonstrated that MsA has an activity peak in the basic pH range (from 7 to 9), so it would properly work in a blood pH of about 7.4. Since the estimated MsA pI is 5.98, under blood physicochemical parameters the protein should show an overall negative charge, and this is also reflected in the active sites’ surroundings. One of the reaction products, aspartic acid, is negatively charged in this neutral to basic pH, so it is repelled by the active site, rapidly liberating it and allowing a new catalysis cycle to occur. This electrostatic effect is well- known and studied for lipases and esterases but it may also apply to hydrolases that show better activity in basic pHs in which one of the reaction products is a negatively charged compound under physiological conditions.^45,46^ Concerning the reaction temperature, MsA showed higher activity at 37 °C, the human body normal temperature, but it retained about 75% activity up to 55 °C and 50% at 65 °C. Compared to MtA and the *M. gordonae* asparaginase, MsA showed a wider range of activity for both pH and temperature.^42,43^ This could be advantageous during moments when the body’s pH and temperature fluctuate due to acidosis and/or fever events, for example, hampering proper asparaginase function in depleting blood l-Asn.

Another important enzyme feature necessary for a good therapeutic asparaginase is its **kinetic parameters** toward l-Asn. It is important to emphasize that while l-Asn blood concentration is in the 40–80 μM range, l-Gln is about 0.5 mM, mainly due to its important role in amine-transport from tissues to the urea cycle in the liver.^11^ In this condition, a good therapeutic prototype asparaginase should have a low *K*_m_ value (reaching maximal velocity in the low blood l-Asn concentration) and a high *k*_cat_ value (with more product being formed per enzyme molecule in each time interval). During l-Asn hydrolysis, the reaction products (l-Asp and NH_3_) are going to diffuse and be consumed in several metabolic pathways, such as the Krebs cycle anaplerotic reactions and the urea cycle, respectively. This makes enzyme reaction product inhibition improbable to take place, so asparaginase would always be in initial velocity conditions, but with lower l-Asn concentration. So, when analyzing the *K*_m_ of one therapeutic prototype asparaginase one should not only focus on normal blood l-Asn concentrations but also in the final concentration range (<0.2 μM) in which lower amounts of this amino acid are available in blood and are sufficient to induce ALL cell apoptosis through l-Asn starvation^44^

MsA has a lower *K*_m_ value than other mycobacterial asparaginases, but a higher *K*_m_ value than commercial EcA2 and ErA formulations. Future work will focus on identifying residues directly related to the kinetic parameters with subsequent in silico analyses to predict potential variants. These variants will then be characterized in vitro to determine if they exhibit reduced *K*_m_ values while maintaining other naturally interesting characteristics of MsA. Nonetheless, *k*_cat_ demonstrated that MsA and MtA have higher and similar values among them, and that they are slightly higher than those from the commercial preparations.^42,43,47,48^ This shows once more that mycobacterial asparaginases are interesting prototypes for the development of therapeutic asparaginase.

Another desirable feature for a therapeutic asparaginase is the induction of minimal **immunogenicity** in human patients, leading to decreased toxic side effects due to immunoreactivity through activation of B and T cells. Additionally, antibody development against therapeutic asparaginase may reduce its half-life inside the human body, requiring a more frequent administration protocol. In extreme cases, this can lead to treatment failure and the necessity for asparaginase formulation swap.^49^ PEGylation is an alternative to reduce immunogenicity, thus increasing biopharmaceutical half-life through lower protein depuration (turnover) and lower immunogenicity.^50^

MsA, like MtA,^42^ has similar numbers of B and CD4+ T cell epitopes predicted within its primary protein sequence. It is important to mention that B cell epitopes require high solvent accessibility to interact with the B cell receptor (BCR), activating signaling cascades and production of an antitherapeutic asparaginase antibody, that may act neutralizing and/or opsonizing the antigen, decreasing enzyme half-life. For CD4+ T cells epitopes, which are first processed and exposed in antigen-presenting cells (APC) through the membrane MHC type-II receptor, less solvent exposed epitopes may have greater impact on immunogenicity.^51^ Nonetheless, all of these epitopes are interesting targets for alteration, creating mutant MsA to be tested for lower immunogenicity, while preserving its already remarkable biochemical features, such as the wide range of reaction pH and temperature, as well as its high *k*_cat_ and considerably low *K*_m_ values.

Finally, and the most controversial topic regarding its therapeutic use is the importance and deleterious effects of the secondary **glutaminase activity** present in several asparaginases. The clinical relevance of this secondary glutaminase activity is still being contested, however many of the toxic side effects of l-asparaginase therapy can be attributed to the l-glutaminase activity, emphasizing the relevance of the search for novel natural asparaginases or efforts to engineer existing ones. Protein engineering is based on a detailed understanding of the structural basis involved in substrate recognition and differentiation by l-asparaginases, both by differential binding (relative to *K*_m_) or catalysis *per se* (relative to *k*_cat_).

Good examples are observed in EcA2 and ErA, targets of direct *in silico* and bench mutagenesis studies. In the former, replacement of Asp248 affected l-Gln turnover much more strongly than l-Asn hydrolysis. *In silico* studies suggested that the selective reduction of glutaminase activity results from small conformational changes that affect active-site residues and catalytically relevant water molecules.^52^ For ErA, an E63Q mutation is thought to hinder the correct positioning of l-Gln but not l-Asn. Substitution of Ser254 with both l-Asn and l-Gln increases the specificity toward l-Asn, but only together with the E63Q mutation. The A31I mutation reduces the substrate *K*_m_ value, a key property to allow the required therapeutic l-Asn depletion. Significantly, an ultralow l-glutaminase ErA variant maintained its cell killing ability.^47^

Another interesting case is that of *Wolinella succinogenes* asparaginase (WoA). In 1970, WoA was described as a glutaminase-free asparaginase that does not induce patient hepatotoxicity and immunogenicity.^53−55^ Around 2000,^56^ this enzyme was produced in large scale, but this formulation showed to be toxic to patients and had detectable glutaminase activity. After genome and protein sequencing, it was observed that glutaminase-free WoA had a serine residue at position 121 (WoA-S121), whereas the one harboring this secondary activity had a proline at this same position (WoA-P121). So, in 2017, Nguyen et al.^11^ showed that both proteins have the same kinetic parameters toward l-Asn, but WoA-S121 has no detectable glutaminase activity. Authors explained this in a three-way logic: (1) l-Asn, when bound in the active site, has a hydrogen bond network capable of stably closing the N-terminal loop that contains two important catalytic residues (Y27 and T14, in WoA); (2) l-Gln, due to its higher volume, causes steric hindrance, preventing loop closure, as it also does not have the same hydrogen bond network of l-Asn able to induce loop closure; (3) l-Asn by itself can force loop closure (leading to the close/active form of asparaginase), whereas l-Gln needs other residue interactions to close it, given by the P121 interaction with Y27 (CH/π interactions between hydrogen δ 1 in P121 and the aromatic ring of Y27).

Primary protein sequence alignment of MsA, MtA, and WoA showed discrepancies in the positions of catalytic residues of both triads within the primary structure. The MsA 3D model created by comparative modeling shows that all important catalytic residues were spatially “correctly” positioned and present in the MsA active sites. As described for MtA,^57^ WoA-Y27 is substituted for MsA-Y276.B, from the other monomer, and so for other important catalytic residues, such as the acidic residue. As described for WoA, l-Asn can maintain a similar hydrogen bond network in the MsA structure, corroborating its ability to solely close the N-terminal loop. As observed, the l-Asn lateral group interacts with MsA-T15, a residue within the N-terminal loop, diminishing the overall loop movement, maintaining it in a closed/active form, and enabling a proper catalysis cycle to occur. Once l-Asp is formed, it does not interact anymore with MsA-T15, thus allowing loop opening and product ejection, with an electrostatic component participation (like the lipase/esterase “electrostatic catapult”.^46^ Similarly, l-Gln also confers steric hindrance within the MsA active site, thus needing the extra proline-tyrosine interaction to stabilize N-terminal loop closure and MsA maintenance in the closed/active form to perform its glutaminase activity. But, as native WoA-S121, MsA has a serine in its 119 position, not interacting with Y276.B. With that, native MsA is thought to be structurally adapted to have a nondetectable glutaminase activity.

These results are corroborated by molecular dynamics, in which MsA-S119 has a larger global movement of the N-terminal loop, indicating that it does not stabilize in its closed/active form, whereas less movement in this protein region is observed in the MsA-P119 mutant. Analyzing interaction energy, it is detectable that in MsA-P119 this proline residue has a larger interaction with other residues when comparing to the serine in the same position in MsA-S119, indicating that the former is interacting with residues (Y276.B and P119, for example), enabling N-terminal loop closure.

Mycobacterial asparaginases are long known for their glutaminase-free feature. Whether this is a desirable characteristic for a therapeutic asparaginase is still under debate. For MsA, some advantages are worth noting, like its low *K*_m_ and high *k*_cat_ toward l-Asn among other already described mycobacterial asparaginases. Additionally, its wider reaction pH and temperature range indicates it can be a useful biopharmaceutical throughout day variance of these variables in the human body, maintaining good activity (over 50%) regardless of the situation. And, like most other mycobacterial asparaginases, MsA has nondetectable glutaminase activity, a path to minimize treatment side effects. But further improvements in this enzyme can be performed, mainly focusing on lowering the *K*_m_ value, reaching blood l-Asn relevant levels, as well as optimizing recombinant MsA production, focusing on optimizing codon usage to favor protein production in soluble form. MsA seems to be a promising prototype for the development of a therapeutic asparaginase, justifying further work on its optimization. The use of mycobacterial enzymes as a framework could lead to the production of variant biomolecules with enhanced characteristics, potentially competitive with current clinical asparaginase formulations. This approach could improve kinetic properties as well as immunoreactivity, thereby reducing the number of patient interventions.

## Supporting Information Available

Additional results from the *in silico* MsA analysis (PDF)
