# Supplementary material for: Characterization of Mycobacterium smegmatis Glutaminase-Free Asparaginase (MSMEG_3173)
Source: ACS Omega. 2024 Sep 12;9(38):40214–25. doi: 10.1021/acsomega.4c06459 (PMC11425952; doi:10.1021/acsomega.4c06459)
Supplement: Supplementary file 1 — ao4c06459_si_001.pdf [file ao4c06459_si_001.pdf]

Supporting information for

Characterization of *Mycobacterium smegmatis* glutaminase-free asparaginase (MSMEG_3173)

Paloma Rezende Corrêa†, Marcos Gustavo Araujo Schwarz†*, Deborah Antunes, Sindy Licette Piñero, Marlon Castro Silva, Mayra Mangabeira Crescêncio, Ana Carolina Ramos Guimarães, Wim Maurits Degrave and Leila Mendonça-Lima

Laboratório de Genômica Funcional e Bioinformática, Instituto Oswaldo Cruz, Fiocruz, Rio de Janeiro, Brazil

† These authors contributed equally to this work.

* Correspondence: schwarz@ioc.fiocruz.br

Contents:

Figure S1. Multiple sequence alignment between L-asparaginase of *Mycobacterium smegmatis* (MsA), *Mycobacterium tuberculosis* (MtA) and *Wolinella succinogenes* (WoA). Black and gray filled positions in the sequence alignment represent fully and partially conserved residues, respectively. Gaps are represented by points. Triads I and II are highlighted in corresponding shades of blue and red. The mutation position from proline to serine is marked in yellow, while MsA-R31 is shown in cyan. S2

Table S1. Evaluation of MsA model and WoA template. S3

Figure S2. Comparison between the crystallographic pose from L-Asp and the best-scoring molecular docking pose outcome. The RMSD between calculated pose (pink) and crystallographic pose (blue) was 0.208 Å. S3

Figure S3. Maps of intermolecular interactions between L-Asp and (A) MsA and (B) WoA. 2D interactions between residues Y276.B and MsA (C), and Y27 and WoA (D) are depiction. S4

Figure S4. Maps of intermolecular interactions between S119P-MsA (A, B) and P21S-WoA (C, D). .S5

Figure S5. Interaction energy between MsA-Y276.B/WoA-Y27 and all other protein residues. Only interactions less than zero are displayed. S6

Figure S6. Three-dimensional structure of catalytic site of MsA and WoA bound with L-Asp and L-Asn. Pose of L-Asp in the MsA site derived from docking assays (A) and the WoA was taken of PDB ID: 5K3O (C). Also represented network interactions with substrate L-Asn in MsA (B) and WoA (D). S7

Figure S7. Three-dimensional structure of catalytic site of MsA and WoA bound with L-Asp and L-Glu. Pose of L-Asp in the MsA site derived from docking assays (A) and the WoA was taken of PDB ID: 5K3O (C). Also represented network interactions with L-Glu in MsA (B) and WoA (D) S8

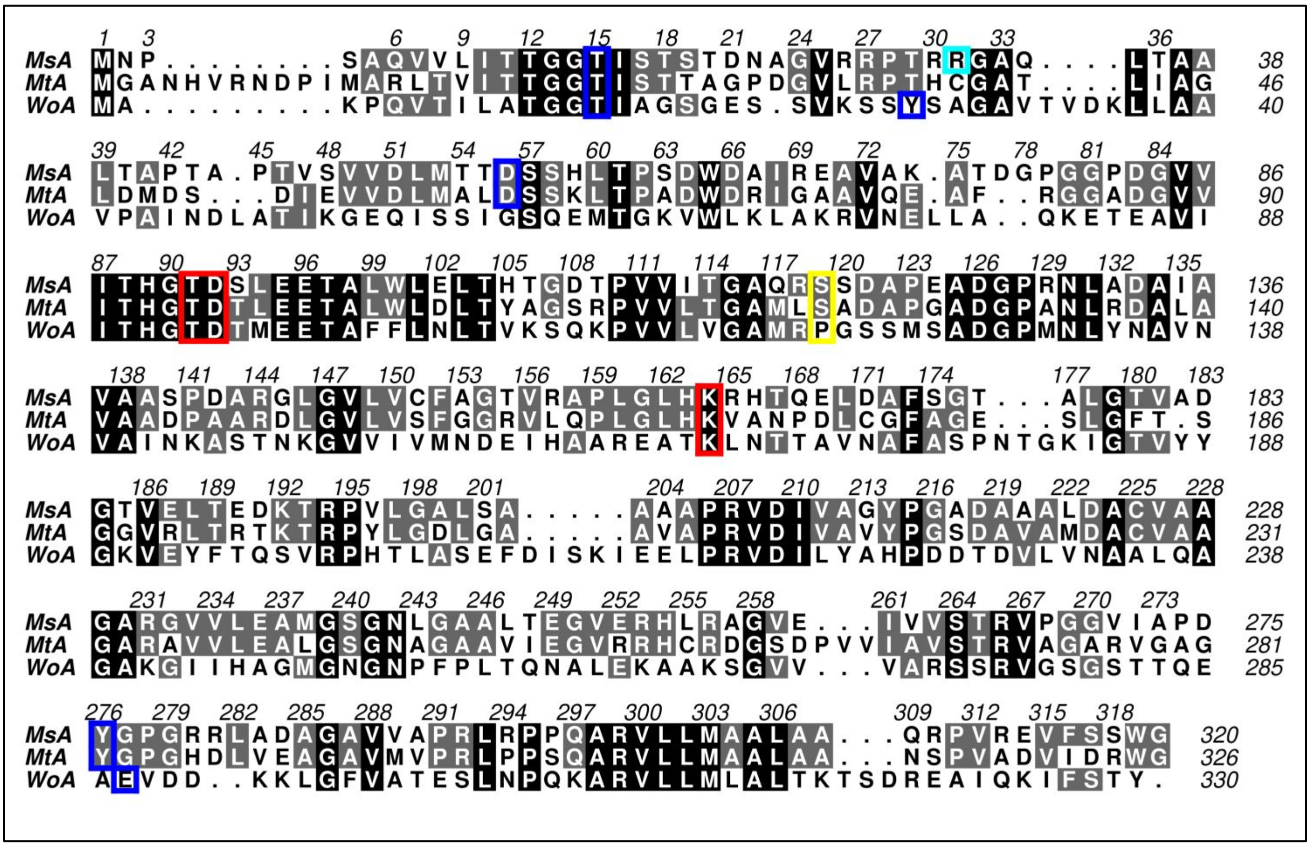

Figure S1. Multiple sequence alignment between L-asparaginase of *Mycobacterium smegmatis* (MsA), *Mycobacterium tuberculosis* (MtA) and *Wolinella succinogenes* (WoA). Black and gray filled positions in the sequence alignment represent fully and partially conserved residues, respectively. Gaps are represented by points. Triads I and II are highlighted in corresponding shades of blue and red. The mutation position from proline to serine is marked in yellow, while MsA-R31 is shown in cyan.

Table S1. Evaluation of MsA model and WoA template.

Assessment	WoA (PDB ID: 5K3O)	MsA model	MsA optimized model
MolProbity Score	1.01	3.06	0.99
Clash Score	1.16	185.25	0.27
Ramachandran Favoured (%)	97.24	95.20	94.26
Ramachandran Outliers (%)	0	1.02	0.94
Rotamer Outliers (%)	1.13	0.11	0.11
QMEAN	0.42	0.74	0.33

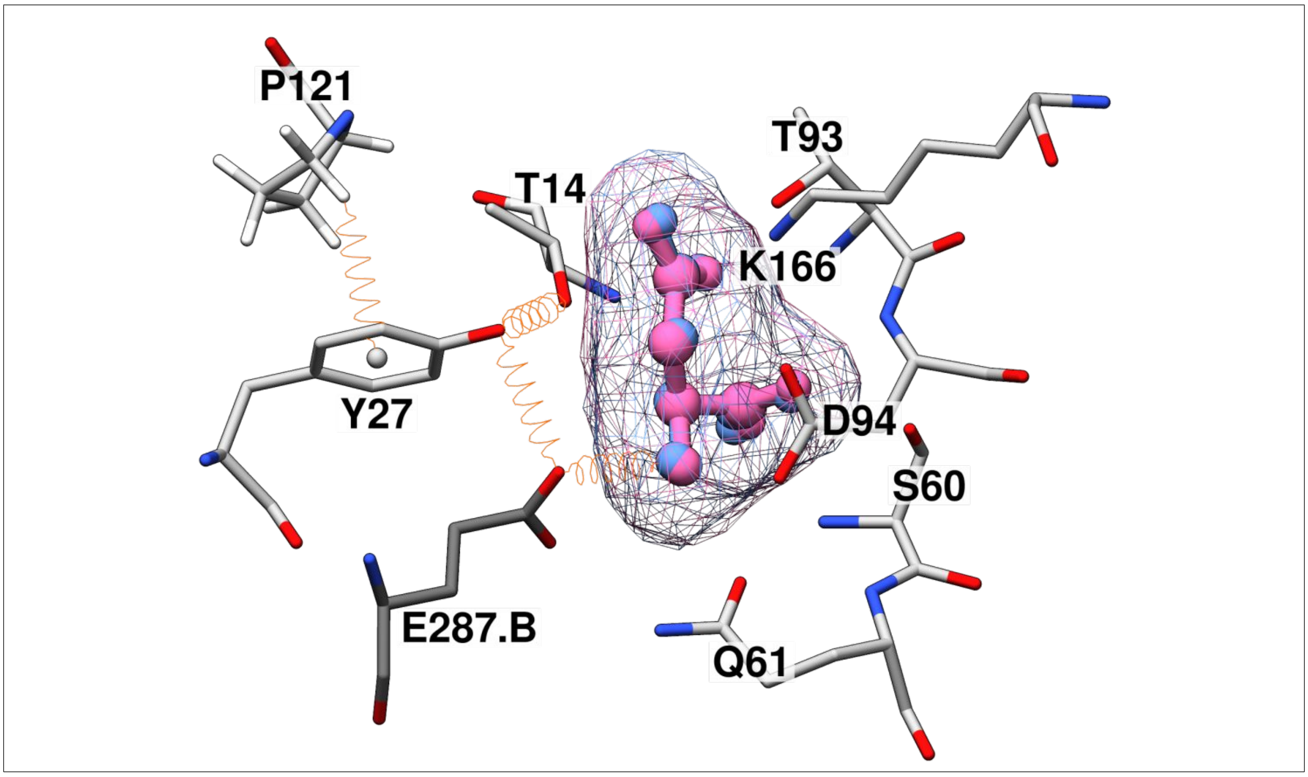

Figure S2. Comparison between the crystallographic pose from L-Asp and the best-scoring molecular docking pose outcome. The RMSD between calculated pose (pink) and crystallographic pose (blue) was 0.208 Å.

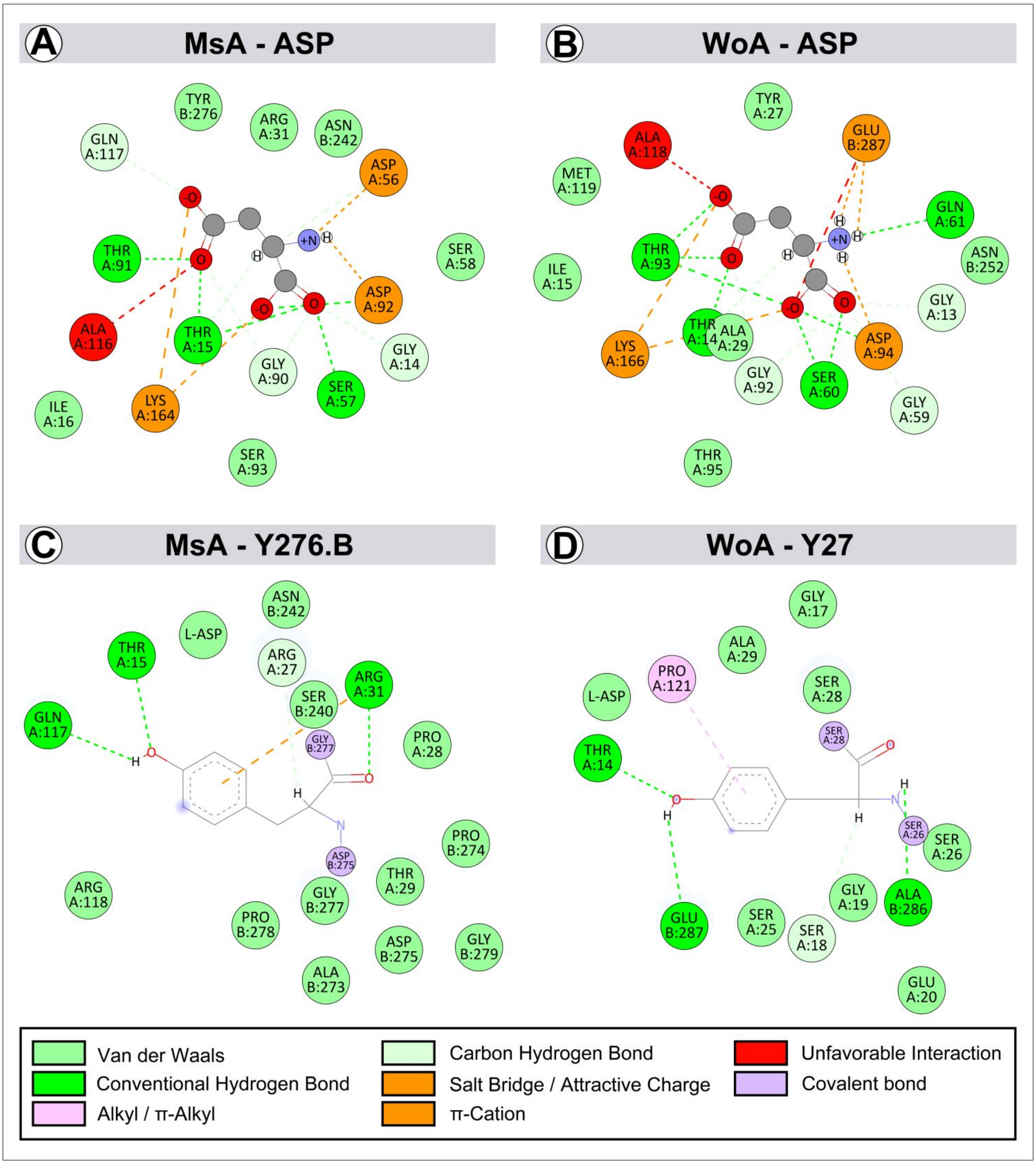

Figure S3. Maps of intermolecular interactions between L-Asp and (A) MsA and (B) WoA. 2D interactions between residues Y276.B and MsA (C), and Y27 and WoA (D) are depiction.

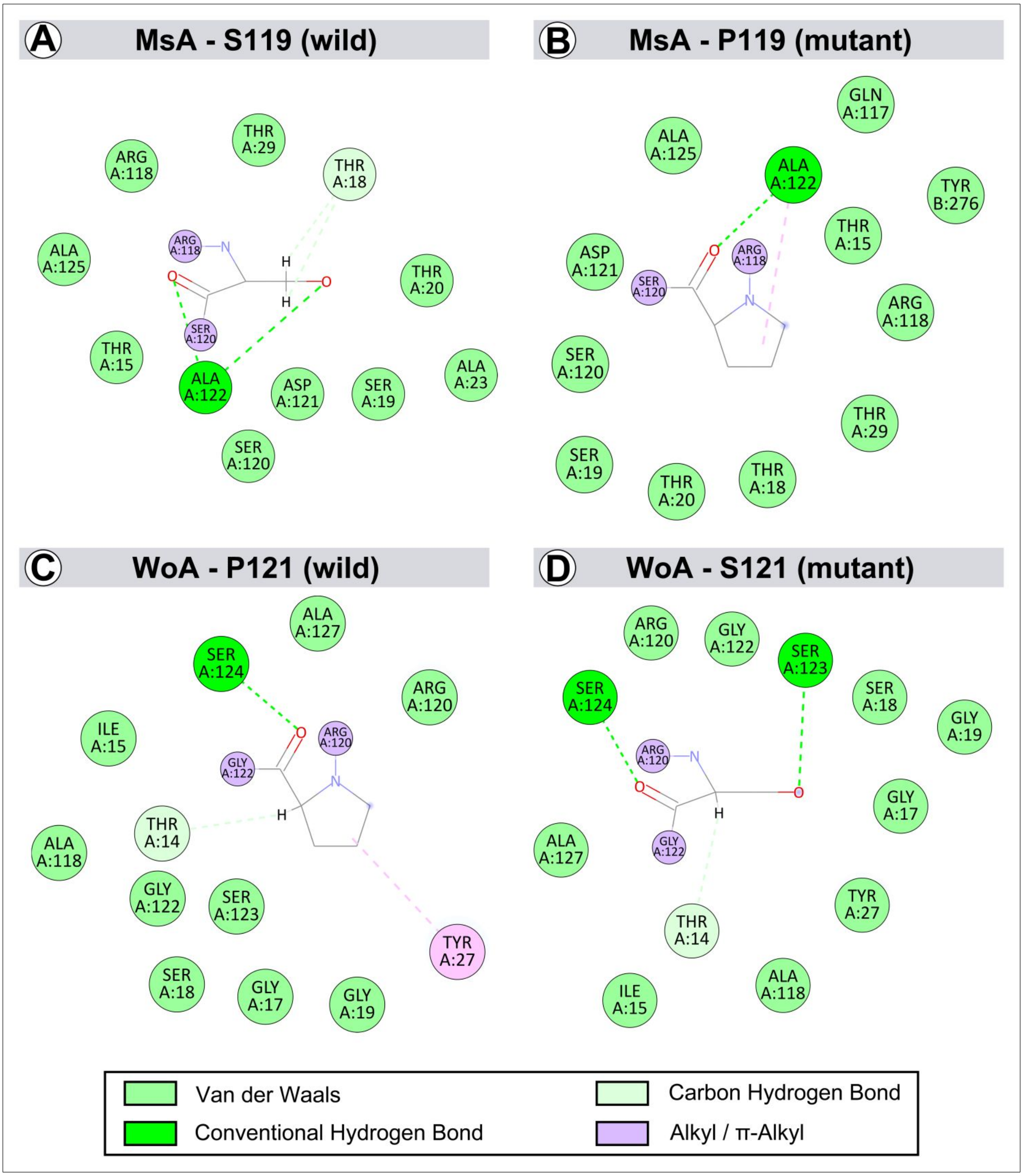

Figure S4. Maps of intermolecular interactions between S119P-MsA (A, B) and P21S-WoA (C, D).

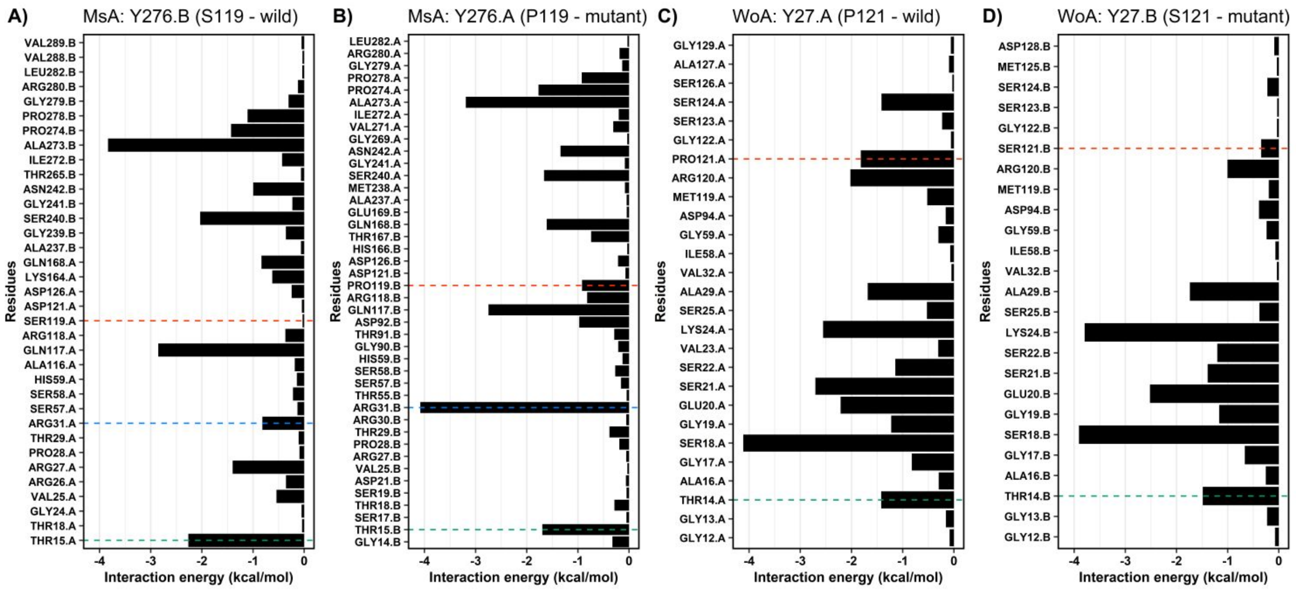

Figure S5. Interaction energy between MsA-Y276.B/WoA-Y27 and all other protein residues. Only interactions less than zero are displayed.

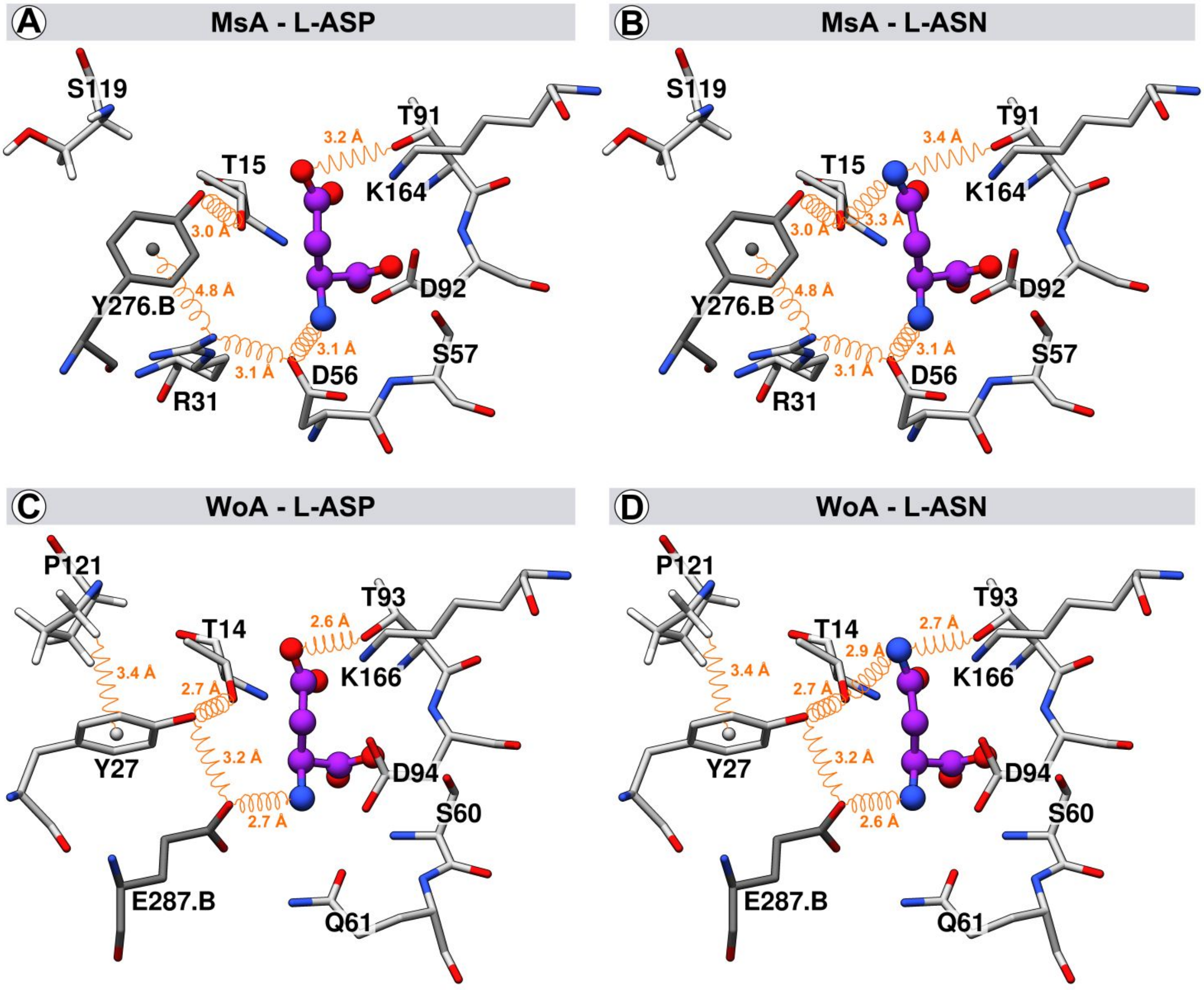

Figure S6. Three-dimensional structure of catalytic site of MsA and WoA bound with L-Asp and L-Asn. Pose of L-Asp in the MsA site derived from docking assays (A) and the WoA was taken of PDB ID: 5K3O (C). Also represented network interactions with substrate L-Asn in MsA (B) and WoA (D).

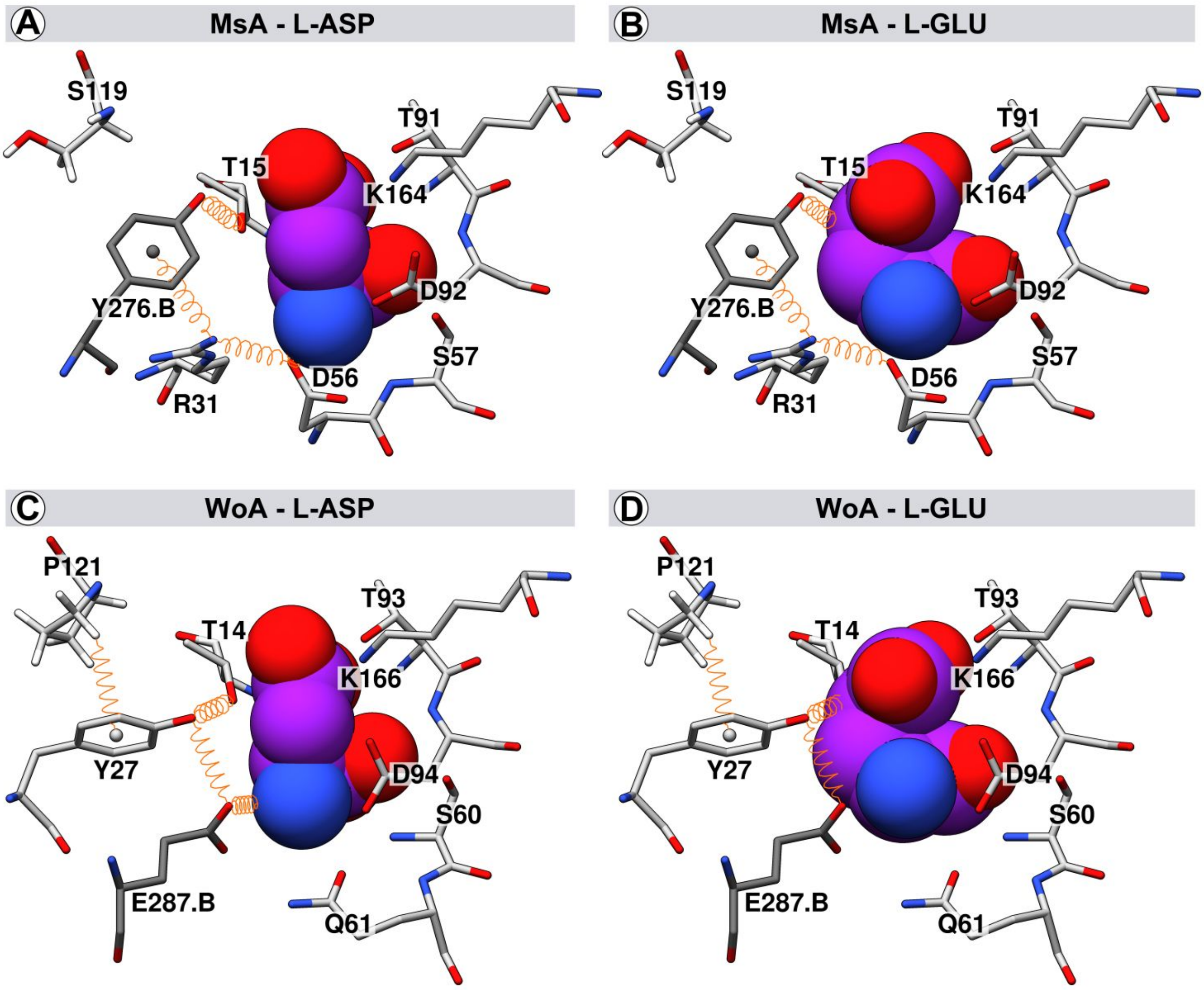

Figure S7. Three-dimensional structure of catalytic site of MsA and WoA bound with L-Asp and L-Glu. Pose of L-Asp in the MsA site derived from docking assays (A) and the WoA was taken of PDB ID: 5K3O (C). Also represented network interactions with L-Glu in MsA (B) and WoA (D)
